# Primary Care Provider Perspectives on Depression Management in Older Adults with Multimorbidity

**DOI:** 10.1093/geroni/igaf122.1565

**Published:** 2025-12-31

**Authors:** Irina Mindlis, Dimitris Kiosses, M Carrington Reid

**Affiliations:** Rutgers University, New York, New York, United States; Weill Cornell Medicine, New York, New York, United States; Weill Cornell Medicine, New York, New York, United States

## Abstract

Primary care remains the main setting for depression treatment among older adults with multimorbidity (OAMM), who are also more likely to experience persistent depression. We sought to characterize primary care providers’ (PCPs) perceived barriers to effective depression management for OAMM receiving primary care. We conducted a cross-sectional survey of 100 PCPs across New York City metro area health systems on satisfaction with resources, barriers, use of evidence-based approaches, and confidence in treating depression in OAMM. Descriptive statistics were summarized using SPSS. Most respondents (58%) were somewhat to very dissatisfied with available resources to manage depression in OAMM. Confidence levels in managing depression in this target population were high, yet rates of measurement-based care for depression were low, with only 19% using validated questionnaires to track symptom trajectories. Competing priorities and limited time within visits were noted as important barriers to depression management in OAMM. PCPs overwhelmingly endorsed a need for better access to mental health providers (85%), especially providers accepting Medicare/Medicaid, and those who offered long-term treatment. Even in the high-resource context of New York City, an area with one of the highest rates of mental health providers in the country, PCPs were dissatisfied with their ability to manage depression in OAMM, reported poor rates of measurement-based care for depression, and advocated for improved access. Future studies with national samples are needed to understand the landscape of depression management for OAMM in primary care. Our findings highlight the need for interventions to improve depression management for OAMM in primary care.

